# Identification of nutritional risk factors and construction of a nomogram prediction model in AIDS patients

**DOI:** 10.3389/fnut.2026.1807908

**Published:** 2026-04-07

**Authors:** Pengpeng Wang, Li Jiang, Xixi Cai, Xueling Huang, Luting Xiao, Jiayi Zhang, E. Liang, Jieli Zhou, Yingling Wang

**Affiliations:** 1 Nursing College of Guangxi Medical University, Nanning, Guangxi, China; 2 Department of Nursing, The First Affiliated Hospital of Guangxi Medical University, Nanning, Guangxi, China

**Keywords:** AIDS, factors, nomogram, nutrition, nutritional risk

## Abstract

**Objective:**

To investigate the nutritional risk factors in AIDS patients and to develop a nomogram prediction model.

**Methods:**

A total of 110 AIDS patients were enrolled between March 2025 and February 2026. Nutritional risk was screened using the Nutritional Risk Screening 2002 (NRS 2002). According to the scores, patients were divided into a well-nourished group (*n* = 81) and a nutritional-risk group (*n* = 29). Clinical data were collected. Variables were first screened by univariable analysis and then entered into a binary logistic regression model to identify nutritional risk factors, with odds ratios (ORs), 95% confidence intervals (CIs), and *p* values reported. Based on the multivariable results, a nomogram prediction model was constructed using R version 4.2.1, and its discriminatory ability was evaluated by the area under the receiver operating characteristic curve (AUC).

**Results:**

Multivariable logistic regression analysis showed that low body mass index (BMI) (OR = 0.654, 95% CI 0.488–0.877, *p* = 0.005), low CD4^+^ T-lymphocyte count (OR = 0.990, 95% CI 0.981–0.999, *p* = 0.031), and low serum albumin level (OR = 0.795, 95% CI 0.689–0.919, *p* = 0.002) were nutritional risk factors in AIDS patients. A nomogram model, constructed based on these variables, demonstrated good predictive performance, with an AUC of 0.959. At the optimal cutoff value of 0.7684, the sensitivity was 79.31% and the specificity was 97.53%.

**Conclusion:**

Low BMI, low CD4^+^ T-lymphocyte count, and low serum albumin are nutritional risk factors in AIDS patients. The nomogram model based on these three indicators shows acceptable predictive accuracy and may have potential value for the early clinical identification of nutritional risk, although further validation is needed.

## 1 Introduction

Acquired Immunodeficiency Syndrome (AIDS) is a chronic infectious disease caused by the human immunodeficiency virus (HIV), characterized by a weakened immune system (1). It severely threatens human health and can even endanger the lives of those infected (2). According to a report by the United Nations Program on HIV/AIDS (UNAIDS), by 2024, approximately 40.8 million people worldwide were living with HIV, with 1.3 million new infections (3). Following the onset of AIDS, the immune system is damaged, and patients often experience symptoms such as difficulty swallowing due to oral fungal infections, leading to reduced appetite and decreased nutrient intake (4, 5). HIV infection increases the metabolic rate, raising energy expenditure and increasing the need for protein and micronutrients to support and improve the compromised immune system (6, 7). Studies have shown that during the asymptomatic phase of AIDS, caloric needs can increase by 15–20%, and once symptoms appear, caloric requirements may rise by 25–40% (8). Throughout the progression of AIDS, patients may experience symptoms such as indigestion, vomiting, and diarrhea. The virus also damages the intestinal barrier, leading to impaired nutrient absorption and increased excretion, which in turn results in nutrient deficiencies and disruptions in acid–base balance (9–11). The relationship between AIDS and nutrition is bidirectional, with both negatively impacting the immune system, increasing the risk of opportunistic infections, and raising the morbidity and mortality rates. Additionally, malnutrition may reduce the effectiveness of antiretroviral therapy (ART) (12, 13).

However, there is limited research on nutritional risk factors in AIDS patients, and the conclusions are inconsistent. Clinical nutritional risk screening tools, such as the Nutritional Risk Screening 2002 (NRS 2002), are suitable for general hospitalized populations but do not fully account for HIV-related factors. Therefore, this study aimed to analyze nutritional risk factors in AIDS patients, develop and validate a nomogram prediction model, and provide evidence-based support for clinical management strategies, optimizing nutritional support pathways, and improving the overall quality of integrated care.

## 2 Materials and methods

### 2.1 Patient selection

Patients diagnosed with HIV/AIDS who meet the inclusion criteria were recruited from the Department of Infectious Diseases, The First Affiliated Hospital of Guangxi Medical University, between March 2025 and February 2026.

### 2.2 Inclusion and exclusion criteria

Inclusion Criteria: (1) Patients diagnosed with AIDS according to the Diagnostic Criteria for HIV/AIDS (WS 293–2008) (14); (2) Age ≥18 years; (3) Patients were fully informed about the study, and written informed consent was obtained.

Exclusion criteria: Patients with severe comorbidities, acute infections, severe mental or cognitive disorders, inability to understand the informed consent form, use of treatments that affect nutritional status, or pregnancy or breastfeeding were excluded. In addition, individuals with missing data for key variables were excluded.

### 2.3 Nutritional screening tool

**Figure 1:**
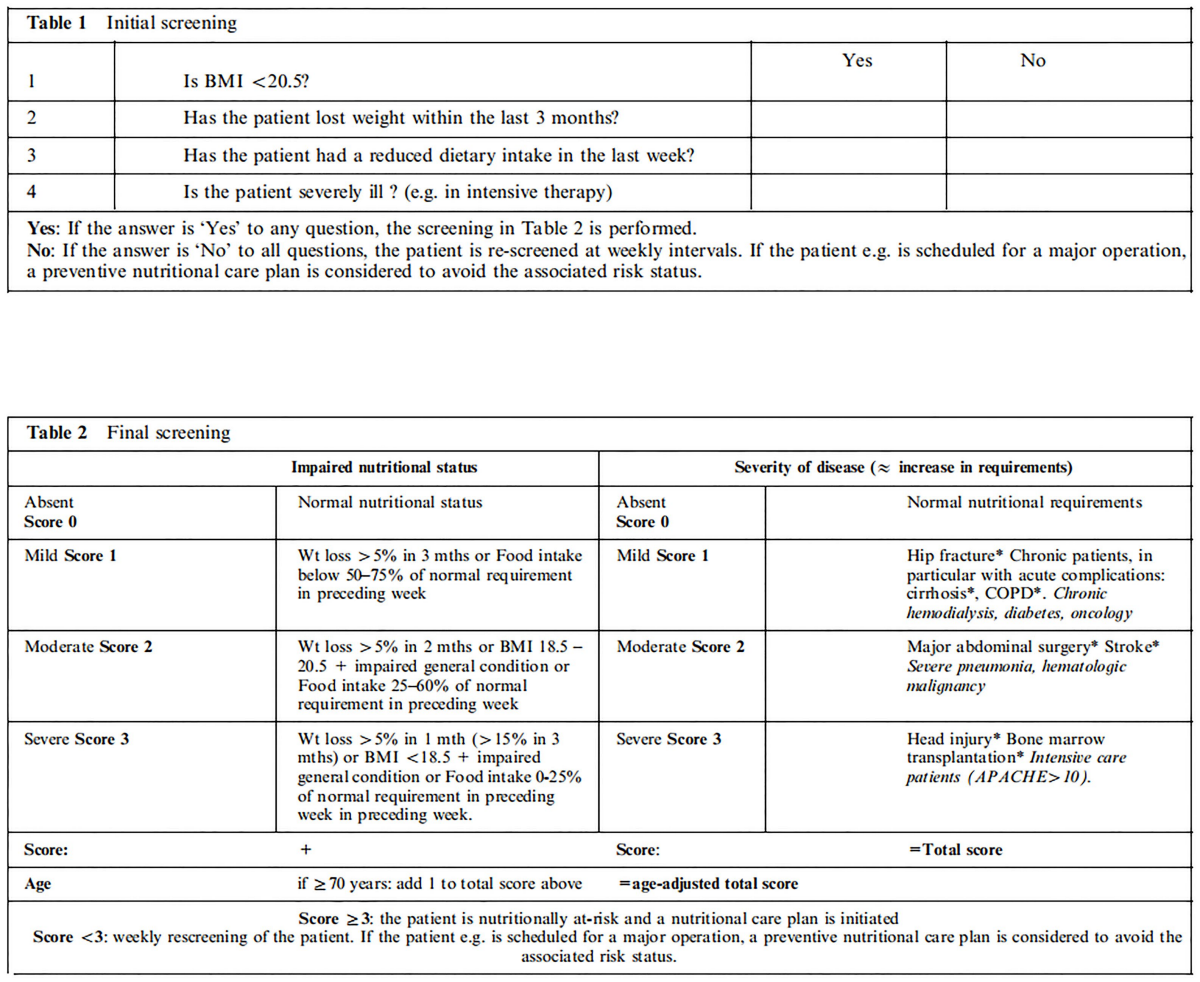
NRS 2002. BMI, body mass index; Wt, weight; COPD, chronic obstructive pulmonary disease; APACHE, acute physiology and chronic health evaluation.

The study used the Nutritional Risk Screening 2002 (NRS 2002) (Figure 1) as the research tool. NRS 2002 (15) consists of two stages: an initial stage with four questions: BMI < 20.5; weight loss in the last 3 months; reduced food intake in the past week; and the presence of severe illness. If the respondent answers affirmatively to any of these questions, the patient proceeds to the screening stage. This stage evaluates weight loss, BMI, and reduced food intake, scoring from 0 to 3, and also assesses the severity of the disease, considering the current clinical condition and the presence of chronic disease complications with acute exacerbations (e.g., major abdominal surgery, cerebrovascular accidents, traumatic brain injury, or bone marrow transplantation), with a score also ranging from 0 to 3. The total score is obtained based on nutritional assessment and disease severity, with an age adjustment for patients over 70 years old. An NRS 2002 score of <3 indicates no nutritional risk, while a score of ≥3 indicates nutritional risk or significant malnutrition, indicating the need for nutritional support. Based on the NRS 2002 score, patients were categorized into the well-nourished group and the nutritional-risk group.

### 2.4 Data collection and statistical analysis

The study primarily collected data on AIDS patients’ age, gender, education level, BMI, CD4^+^ T lymphocyte count, and blood indicators. Data analysis was performed using SPSS 27.0 and R 4.2.1. Continuous variables were expressed as mean ± standard deviation (x̅ ± s). In univariate analysis, continuous variables were analyzed using the *t*-test, categorical variables were analyzed using the Chi-square (χ^2^) test, and ordinal or non-normally distributed variables were analyzed using the Wilcoxon Rank-Sum test. Variables with a *p*-value < 0.05 were selected for inclusion in the logistic regression analysis to obtain the adjusted odds ratios (ORs) and 95% confidence intervals (CIs). A two-tailed test was used, with *p* < 0.05 considered statistically significant.

After identifying the nutritional risk factors for AIDS patients through multivariable analysis, a nomogram prediction model was constructed based on these risk factors. To evaluate the model’s performance, the area under the receiver operating characteristic (ROC) curve (AUC) and a calibration curve were used.

### 2.5 Statement of ethics

The study was approved by the Ethics Committee of Guangxi Medical University (Approval number: KY20250185).

## 3 Results

A total of 110 AIDS patients were included in the study. Among them, 81 patients were assigned to the well-nourished group and 29 patients to the nutritional-risk group. Univariate analysis showed that BMI (*t* = 7.722, *p* < 0.001), CD4^+^ T lymphocyte count (*t* = 6.848, *p* < 0.001), total protein (TP) (*t* = 2.379, *p* = 0.019), albumin (ALB) (*t* = 9.180, *p* < 0.001), potassium (K) (*t* = 3.796, *p* < 0.001), sodium (Na) (*t* = 2.517, *p* = 0.017), and chloride (Cl) (*t* = 2.274, *p* = 0.030) showed statistically significant differences and were included in the binary logistic regression analysis. The results are shown in Tables 1, 2.

**Table 1:** Results of univariate analysis.

Variable	Well-nourished group (*n* = 81)	Nutritional-risk group (*n* = 29)	*X*^2^/*Z*	*p*
Gender				
Female	15	5	0.023	0.878
Male	66	24		
Education level^*^				
Primary school or below	33	17	−1.149	0.156
Junior high school or high school	35	8		
College or above	13	4		

^*^The Wilcoxon Rank-Sum test was used.

**Table 2:** Results of univariate analysis.

Variable	*t*	*p*
Age (years)	0.149	0.858
BMI (kg/m^2^)	7.722	<0.001
CD4^+^ T lymphocyte count (cells/μL)	6.848	<0.001
TBiL (μmol/L)	−1.035	0.307
DBiL (μmol/L)	−0.965	0.340
IBil (μmol/L)	−1.595	0.114
TP (g/L)	2.379	0.019
ALB (g/L)	9.180	<0.001
GLO (g/L)	−0.101	0.919
GGT (μ/L)	1.648	0.103
TBA (μmol/L)	−0.500	0.618
AST (μ/L)	−0.261	0.795
ALT (μ/L)	0.527	0.599
ALP (μ/L)	0.853	0.396
PA (mg/L)	1.945	0.054
UREA (mmol/L)	−0.200	0.984
CREA (μmol/L)	0.586	0.559
UA (μmol/L)	0.755	0.452
K (mmol/L)	3.796	<0.001
Na (mmol/L)	2.517	0.017
Cl (mmol/L)	2.274	0.030
Ca (mmol/L)	1.034	0.303

BMI, body mass index; TBiL, total bilirubin; DBiL, direct bilirubin; IBil, indirect bilirubin; TP, total protein; ALB, albumin; GLO, globulin; GGT, gamma-glutamyl transferase; TBA, total bile acids; AST, aspartate aminotransferase; ALT, alanine aminotransferase; ALP, alkaline phosphatase; PA, prealbumin; UREA, urea; CREA, creatinine; UA, uric acid; K, potassium; Na, sodium; Cl, chloride; Ca, calcium.

Binary logistic regression analysis revealed that low BMI (OR = 0.654, 95% CI 0.488–0.877, *p* = 0.005), low CD4^+^ T lymphocyte count (OR = 0.990, 95% CI 0.981–0.999, *p* = 0.031), and low albumin (OR = 0.795, 95% CI 0.689–0.919, *p* = 0.002) were nutritional risk factors in AIDS patients. The results are shown in Table 3.

**Table 3:** Results of binary logistic regression analysis.

Variable	*B*	Standard error	Wald	Significance	Exp (*B*)	95% confidence interval for Exp (*B*)	
						Lower limit	Upper limit
BMI (kg/m^2^)	−0.425	0.150	8.069	0.005	0.654	0.488	0.877
CD4^+^ T lymphocyte count (cells/μL)	−0.01	0.005	4.663	0.031	0.990	0.981	0.999
ALB (g/L)	−0.229	0.074	9.679	0.002	0.795	0.689	0.919
TP (g/L)	0.079	0.049	2.584	0.108	1.082	0.983	1.192
Constant	9.108	3.379	7.264	0.007	9025.521		

BMI, body mass index; ALB, albumin; TP, total protein.

Based on the results of the binary logistic regression analysis, the regression equation was as follows:

$\begin{array}{l} \text{logit}(p)=9.108–0.425\times \mathrm{BMI}-0.010\times \mathrm{CD}4+\\ T\;\text{lymphocyte count}-0.229\times \mathrm{ALB} \end{array}$ .

**Figure 2:**
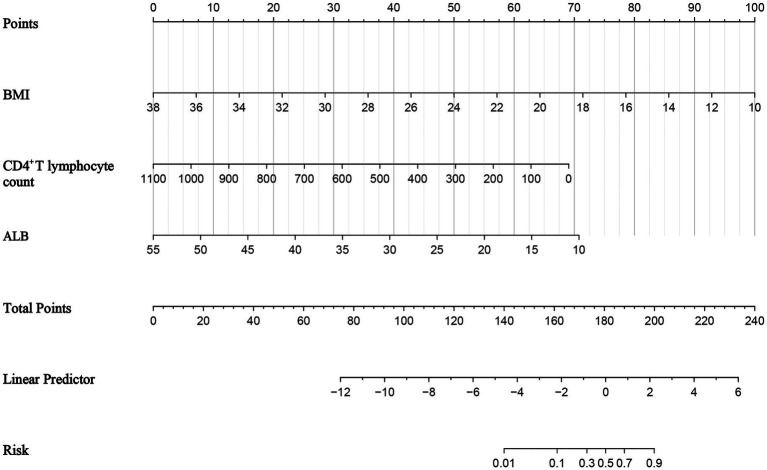
Nomogram prediction model.

**Figure 3:**
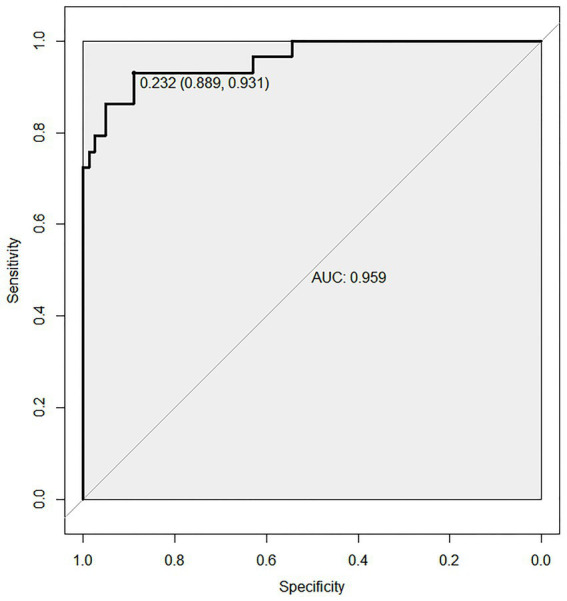
ROC curve of the predictive model.

A nomogram prediction model was constructed using R 4.2.1, as shown in Figure 2. The receiver operating characteristic (ROC) curve was plotted using R 4.2.1 and MedCalc 20.100 software to verify the model’ s predictive performance for nutritional risk in AIDS patients. The predicted probabilities from the modeling set were used as the test variable, and the actual occurrence of nutritional risk (1 = occurred, 0 = not occurred) was used as the state variable. The area under the curve (AUC) and 95% confidence interval (95% CI) were calculated. The optimal cutoff value corresponding to the maximum Youden index was determined to be 0.7684, with a sensitivity of 79.31% and a specificity of 97.53%. The final AUC of the model was 0.959, indicating good discriminatory ability, as shown in Figure 3.

**Figure 4:**
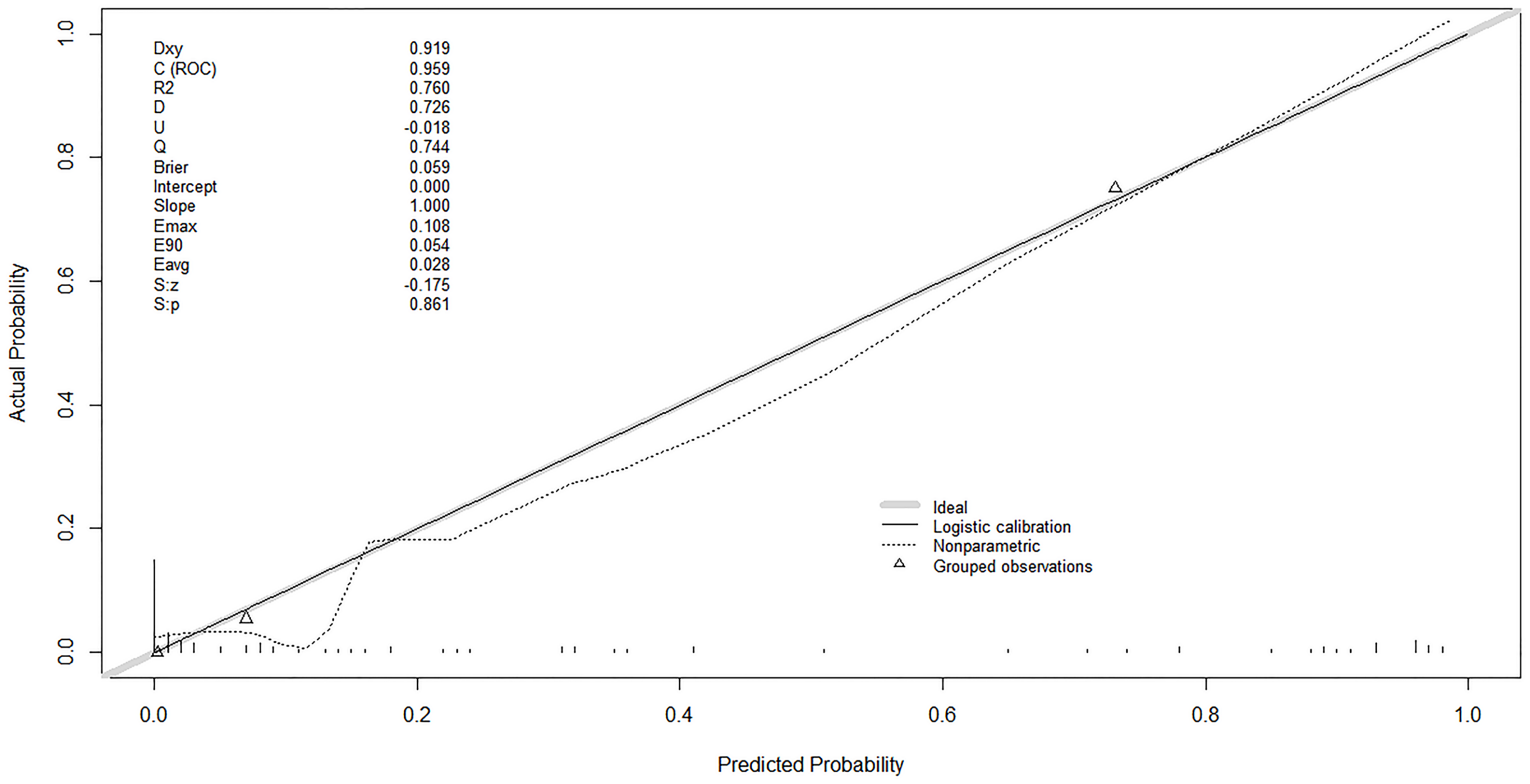
Calibrated curve of the predictive model.

For calibration assessment, the model showed optimal agreement between predicted and observed probabilities (Figure 4). The mean absolute error (Eavg) was 0.028, and the maximum absolute error (Emax) was 0.108, reflecting minimal bias across the entire range of predicted probabilities. The Brier score was 0.059, further confirming the model’s high overall predictive accuracy and good calibration performance. The calibration curve closely followed the ideal 45°line, visually demonstrating the strong alignment between predicted and actual probabilities.

To assess the stability and robustness of the predictive model, bootstrap resampling (B = 1,000) was performed for internal validation using the rms package in R 4.2.1. After bootstrap correction, the Somers’ Dxy coefficient was 0.902, corresponding to a corrected AUC of 0.951 (calculated as (Dxy + 1)/2). The optimism value for Dxy was 0.017, indicating minimal overfitting and confirming the model’s reliable discriminative performance. The bias-corrected calibration metrics further validated this performance: intercept = −0.024 (close to 0), slope = 0.882 (close to 1), and maximum calibration error (Emax) = 0.032. Detailed numerical results of the internal validation are summarized in Table 4.

**Table 4:** Internal validation results of the predictive model.

Metric	Value	Description
Apparent AUC	0.957	Original model performance
Bootstrap-corrected Dxy	0.902	After bootstrap correction
Bootstrap-corrected AUC	0.951	Calculated as (Dxy + 1)/2
Optimism (Dxy)	0.017	Measure of overfitting
Calibration intercept	−0.024	Closer to 0 = better calibration
Calibration slope	0.882	Closer to 1 = better calibration
Maximum calibration error (Emax)	0.032	Smaller value = better calibration

AUC, area under the receiver operating characteristic curve.

**Figure 5:**
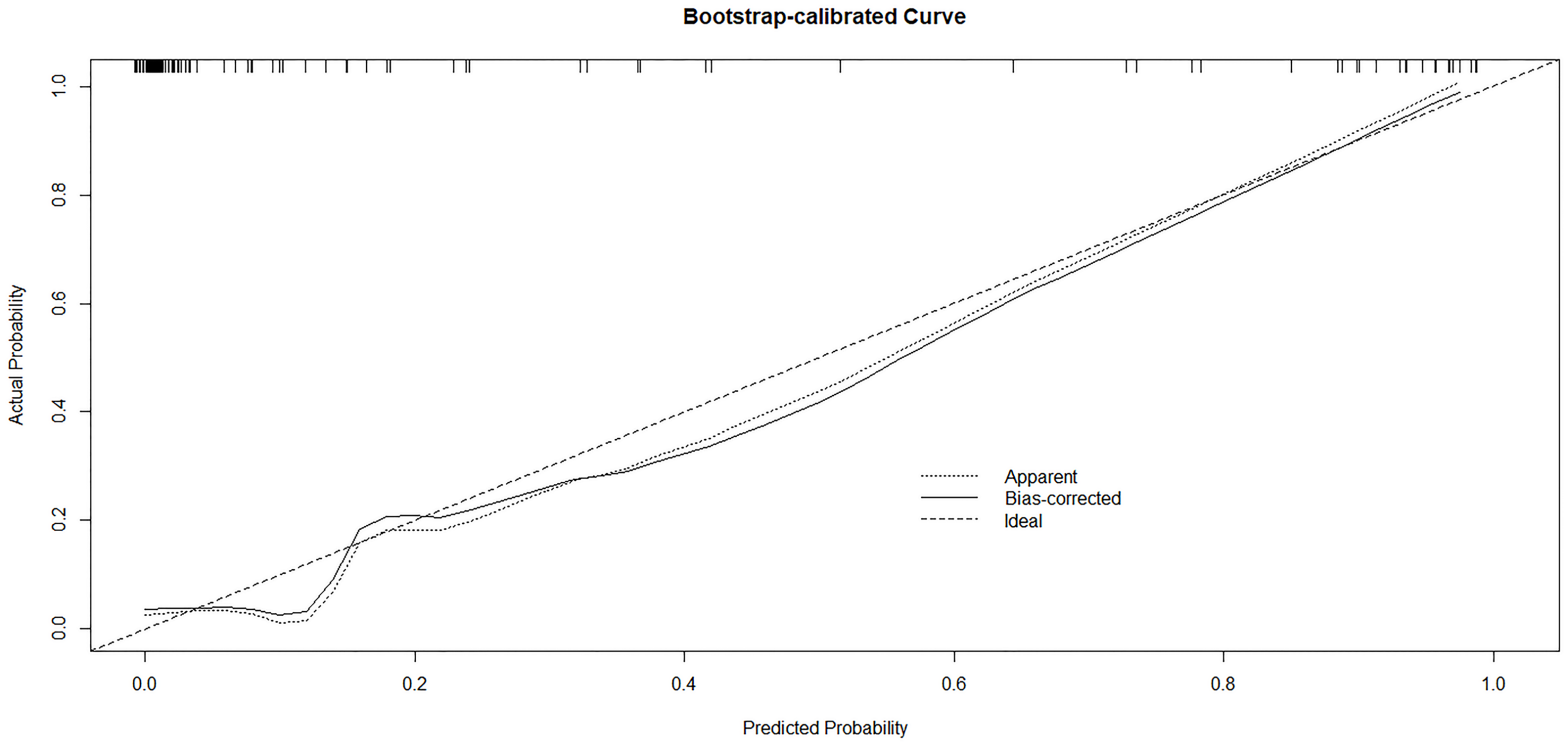
Bootstrap-calibrated curve for internal validation of the predictive model.

For calibration assessment, a bootstrap-calibrated curve was generated (Figure 5), which demonstrated excellent agreement between the predicted probabilities of nutritional risk and the actual observed outcomes.

**Figure 6:**
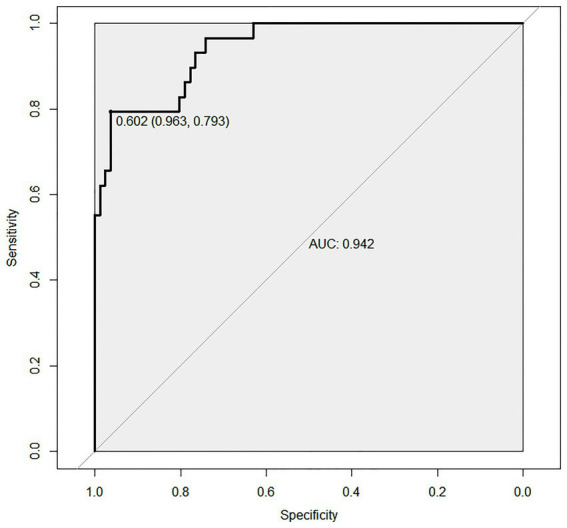
ROC curve for sensitivity analysis (excluding BMI) of the predictive model.

A sensitivity analysis was performed by excluding the BMI variable from the predictive model to assess the robustness of the findings. As shown in the revised Figure 6, the area under the receiver operating characteristic curve (AUC) of the model without BMI was 0.942 (95% CI, 0.793–0.963), which remained at a high level comparable to the full model. This result indicates that the predictive performance of our model is not solely dependent on the BMI variable, and the model exhibits excellent stability and robustness.

**Figure 7:**
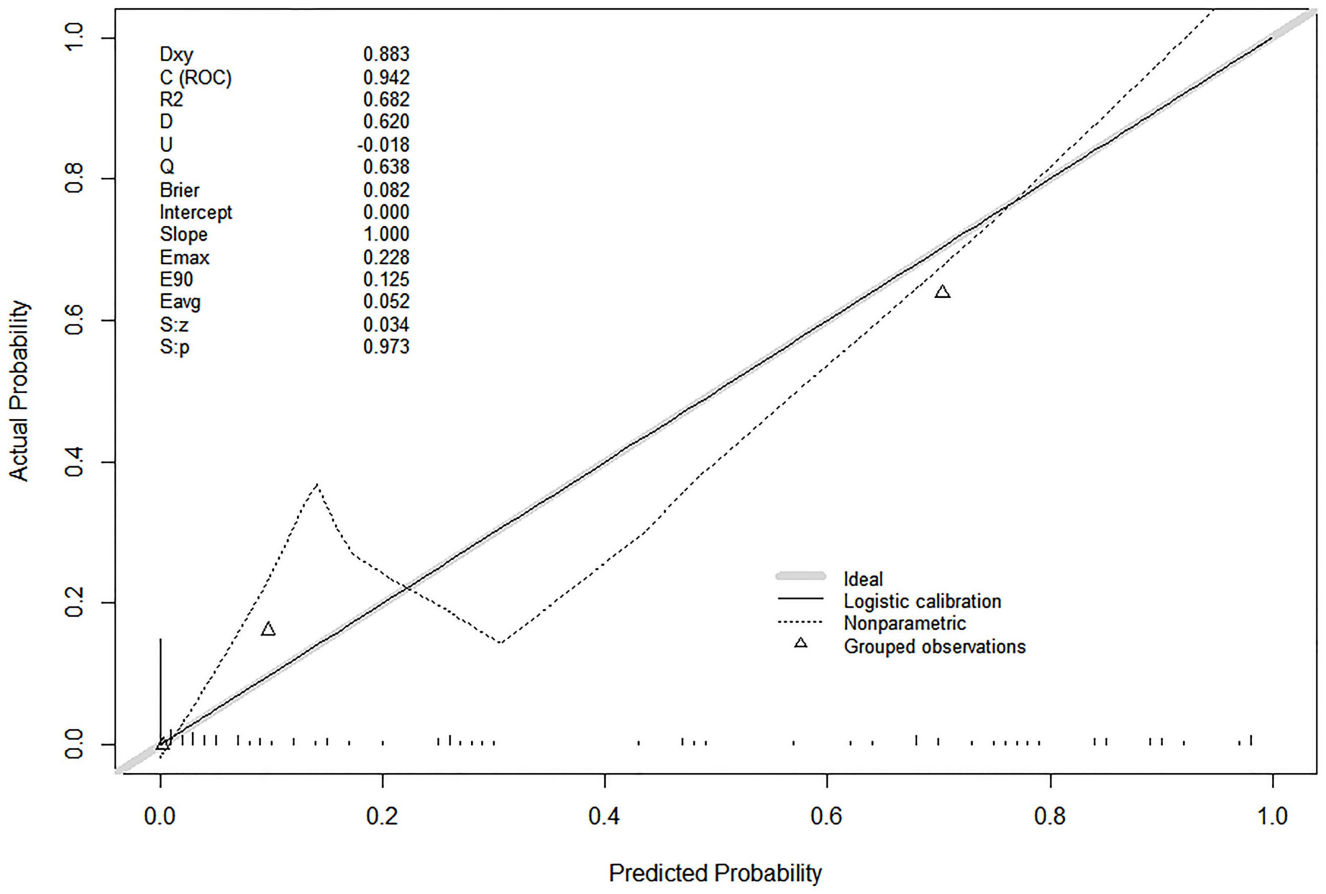
Calibration curve for sensitivity analysis (excluding BMI) of the predictive model.

After excluding BMI, the model still demonstrated excellent calibration: mean absolute error (Eavg) = 0.052, and Brier score = 0.082 (Figure 7). The slight deviation in the nonparametric calibration curve at low predicted probabilities was mainly due to the limited number of events in the low-risk subgroups, which did not affect the overall predictive accuracy of the model.

**Figure 8:**
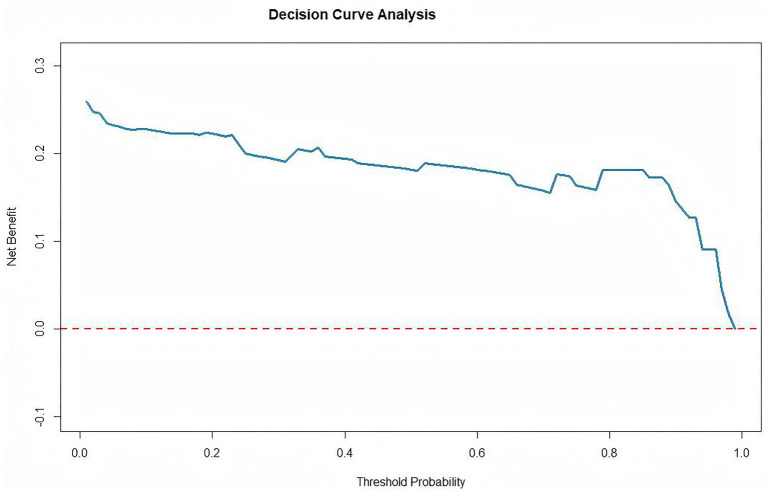
Decision curve analysis of the predictive model.

Decision Curve Analysis (DCA) was performed to evaluate the clinical utility of the predictive model. As shown in the revised Figure 8, the DCA curve demonstrates that the model provides a positive net benefit across a wide range of threshold probabilities (0 to approximately 0.98), which is superior to the strategy of treating no patients (net benefit = 0). This result indicates that using the model to guide clinical decisions would yield meaningful clinical utility.

## 4 Discussion

The study analyzed nutritional risk factors in AIDS patients and constructed a nomogram prediction model, aiming to provide evidence for nutritional support interventions in clinical practice. By performing univariate analysis and binary logistic regression analysis in 110 AIDS patients, the study found that BMI, CD4^+^ T lymphocyte count, and ALB were nutritional risk factors in AIDS patients. The constructed nomogram prediction model demonstrated high predictive performance, with an AUC of 0.959, showing good discriminatory ability.

Firstly, BMI = weight (kg)/height^2^ (m^2^). BMI is an indicator used to assess the nutritional status of adults, and a low BMI indicates a higher risk of insufficient nutrient intake. BMI is commonly used as a screening or assessment parameter in various nutritional screening tools. The NRS 2002 includes a pre-screening step with four questions, one of which is “Is the patient’s BMI < 20.5 kg/m^2^?” (16). BMI is also a standard screening parameter in the Mini Nutritional Assessment (MNA), and the MNA score is positively correlated with BMI (17). The Global Leadership Initiative on Malnutrition (GLIM) includes phenotypic criteria, which define low BMI as <20 kg/m^2^ for individuals under 70 years and <22 kg/m^2^ for individuals over 70 years in non-Asian populations, and <18.5 kg/m^2^ for individuals under 70 years and <20 kg/m^2^ for individuals over 70 years in Asian populations (18). Furthermore, according to the European Society for Clinical Nutrition and Metabolism (ESPEN) consensus, a BMI < 18.5 kg/m^2^ is defined as malnutrition (19). The study found that BMI was consistent with previous research as an independent nutritional risk factor (20).

Secondly, a decrease in CD4^+^ T lymphocyte count is closely related to the impaired immune function in AIDS patients. CD4^+^ T cells are a critical component of the immune system, and a reduction in their number signifies a weakened immune function (21). A decrease in CD4^+^ T lymphocyte count may lead to a weakened intestinal immune barrier, increasing the risk of intestinal infections and inflammation, which further affects the digestion and absorption of nutrients, creating a vicious cycle.

ALB is a sensitive indicator reflecting the nutritional status of the body, as low levels often indicate protein malnutrition. In the study, low ALB levels were significantly associated with increased nutritional risk. Research shows that ALB <35 g/L indicates hypoalbuminemia, and persistent hypoalbuminemia is an important indicator of malnutrition (22). Eckart et al. (23) confirmed that the nutritional risk in adult patients is related to ALB. Additionally, ALB is a visceral protein and a sensitive indicator of marginal nutrient deficiencies. It reflects changes in protein-energy nutrition and is also used as a nutritional status marker in orthopedic patients (24).

Although the NRS 2002 tool has shown good screening effectiveness in a wide range of hospitalized patients, it does not fully account for the specific factors associated with HIV infection. In contrast, the nomogram model in the study, by combining the three variables closely related to the nutritional status of AIDS patients—BMI, CD4^+^ T lymphocyte count, and ALB—achieved an AUC of 0.959 in predicting nutritional risk, showing excellent discriminatory ability. BMI, CD4^+^ T lymphocyte count, and ALB are routine tests in AIDS patients and are easy to obtain, which enhances the convenience and operability of the new model. This has significant clinical implications, particularly for nurses and doctors working on the front lines of clinical practice, who can quickly identify high-risk patients using the model and then implement more effective nutritional interventions.

However, despite the high predictive performance and clinical applicability of the new nomogram model, it still has certain limitations. First, the model was built using patient data from a single hospital, with a relatively small sample size, so its generalizability and external validation need further improvement. In addition, the number of nutritional-risk cases in this study was relatively limited (*n* = 29). The final multivariate regression model included three predictor variables, and the corresponding events-per-variable ratio was approximately 10, which meets the commonly recommended criterion of at least 5–10 events per variable to ensure model stability. Nevertheless, the relatively small number of events may still affect the robustness and generalizability of the model. This should be regarded as a potential limitation, and future studies with larger sample sizes and more events are needed to further validate and optimize the model. Additionally, although the model covers the three key indicators—BMI, CD4^+^ T lymphocyte count, and ALB—there may still be other potential nutritional risk factors that were overlooked. As an indicator of the NRS-2002, the direct correlation between BMI and outcome indicators may objectively enhance the model’s predictive effect on nutritional risk, leading to a certain degree of overestimation of model performance. The original intention of including BMI as a predictor in this study was based on its practical value of being easily accessible in clinical practice and quickly reflecting the patient’s basic nutritional status; moreover, BMI itself is an important clinical indicator related to nutritional risk and disease progression in the AIDS patient population. In future research, we will focus on optimizing the selection of predictors, by incorporating indicators that do not overlap with NRS-2002 (such as inflammatory factors, body composition analysis results, etc.) or adopting other nutritional risk assessment criteria that do not include BMI as the outcome definition, to reduce potential biases caused by such indicator overlap and further improve the scientificity and reliability of the model. Future research could incorporate more clinical variables to further refine the model and improve its predictive accuracy. Future research could incorporate more clinical variables to further refine the model and improve its predictive accuracy.

### 4.1 Clinical value

The study identified that low body mass index (BMI), low CD4^+^ T-lymphocyte count, and low serum albumin level—three readily available routine clinical indicators—are independent nutritional risk factors in patients with acquired immunodeficiency syndrome (AIDS), facilitating clinicians’ early identification of high-risk individuals. Furthermore, the nomogram prediction model constructed using only these three routine, easily accessible parameters is characterized by fewer indicators, high clinical accessibility, and simple operation, enabling rapid nutritional risk assessment. Concurrently, the model demonstrates acceptable predictive accuracy, allowing clinicians to promptly identify AIDS patients at high nutritional risk and initiate timely targeted nutritional support interventions. This provides a practical and convenient assessment tool for individualized nutritional management in AIDS patients.

## 5 Conclusion

Low BMI, low CD4^+^ T lymphocyte count, and low albumin are nutritional risk factors for AIDS patients. The nomogram model constructed based on these three indicators showed acceptable predictive performance. While this model may hold potential for early nutritional risk identification in clinical practice, its validity and generalizability require further validation in multiple centers and regions.

## Data Availability

The raw data supporting the conclusions of this article will be made available by the authors, without undue reservation.
